# An Essay Towards a Pathology of Eruptive Fevers

**Published:** 1820-07

**Authors:** Richard Harrison

**Affiliations:** Fellow of the Royal College of Physicians, &c. &c.


FOR THE LONDON MEDICAL AND PHYSICAL JOURNAL.

*An Essay towards a Pathology of Eruptive-Fevers.* Delivered, as a Gulstonian Lecture, before the Royal College of Physicians of London, in the Year 1820; by RICHARD HARRISON, M.D. F.L.S. Fellow of the Royal College of Physicians, &c. &c.

THE subjects of the following observations are the several eruptive fevers which affect the human body; and my object in taking these diseases into consideration, is to try how far I may be able to elucidate them, by employing physiology as my guide, in an investigation of the nature and order of their phenomena.

It is necessary, in the first place, for me to give an idea of what I consider to constitute fever: not, according to the method of nosologists, what combination and series of symptoms should be thus termed; but what is its physiological nature, according to the analytical method I shall adopt in treating of the forms of that affection which are the especial subjects of the following discourse.

Fever I consider to be a derangement of certain functions of the body, dependant on irritation of some particular part, which becomes a cause of disturbance to the rest of the system, by means of the sympathy existing between its several organs. But, irritation of any part will not give rise to fever; and it appears, that it is irritation of one organ solely that will produce this effect, and that organ is the mucous membrane.

The functions deranged in fever are those of the secretory organs: hence the morbid state of the secreted fluids, and the alteration of the temperature of the body. From the derangement of these functions arise all the essential phenomena of fever. The increased frequency of the action of the heart and arteries, and the disturbance of the intellects, which ordinarily accompany it, are accidental phenomena, and are not necessary in order to constitute fever. The intellects are often not disordered; and we not unfrequently observe typhous fever run its whole course without any increased frequency of the pulse. These are accidental sympathetic phenomena, and their origin will be hereafter explained, when I come to treat particularly of diseases dependant on sympathy with some other morbid states.

The whole of the secretory organs are lined with mucous membranes;* and irritation of some portion of those membranes is constantly present in fever. The portion of the mucous membranes commonly first, and most generally, if not always, affected, is that of the upper part of the alimentary canal: hence arise the furred tongue, thirst, nausea, vomiting, and anxiety; and these are generally amongst the first order of the phenomena of fever.

Fever does not immediately result from irritation of any other part. We find extensive and very severe inflammation of serous and cellular membranes, and of the fibrous and bony structures, and no fever exists: but, if the irritation of any of those parts be participated by the mucous membranes, all the phenomena of fever immediately appear.

---

* The skin, everywhere continuous on the exterior of the body, is reflected through different internal openings, and gives rise to the mucous membranes. The limits of each of these systems is shown by a red line. The interior of this line marks the commencement of the mucous surfaces; whilst the dermoid texture is placed without it. Still the mucous membranes and skin are not so distinct from each other in respect to organization, as in regard to colour; and I shall perhaps speak more correctly, if I say, that each system terminates in the other by insensible gradations.

The chief prolongation of the skin into the mucous membranes, is that which enters by the nose and mouth, and lines throughout the facial sinuses and the bronchiæ, as well as the whole course of the primæ viæ, giving off, in its passage, portions to the excretory ducts of all the secretory organs that terminate in the alimentary canal. There are two smaller divisions, the one lining the genital and urinary organs, and the other the ducts of the mammillary glands.

Further observation will perhaps prove, that the functions of the mucous membrane of the alimentary canal are always disordered in fever. The stomach and small intestines appear to be the seat of local disease in continued fevers. The tongue, which sympathizes so intimately with the digestive organs, is either covered by a morbid secretion; or it is florid, tumid, and devoid of the natural secretion, when the irritation is intense in degree. There is always some degree of nausea and sense of anxiety about the stomach, and generally want of appetite for food. In some cases, however, there is an increased desire for food, especially at the onset of the disease; but this marks a state of irritation of the stomach, that, on becoming more violent, produces nausea, vomiting, and total loss of appetite. The most strongly-marked cases of inflammation of the mucous membrane of the stomach often commence in the way just indicated. The patient at first suffers an inordinate craving for food, which is not immediately removed by the introduction of aliment into the stomach; it, however, usually subsides in about half an hour or so; but it re-appears soon afterwards with increased violence, and in a short time, as a few days, especially if stimulating food is taken, it becomes a sense of absolute pain, attended with nausea, vomiting, and disgust for every kind of alimentary matter, except cool and unirritating liquids. This has often been observed in yellow-fever, the origin of which is one of the most severe forms of inflammation of the mucous membrane of the stomach and duodenum. The disorder of the stomach above described is not unfrequently the only sign of the approaching disease for one or two days; and we may often witness the same circumstances in the several forms of fever that occur in our own climate.

It seems, also, that intermittent fevers are as constantly related to disorder of the mucous membrane of the large intestines. The connexion of dysentery with fever of this type has been long and frequently observed; and many other circumstances tending to show the connexion above mentioned, might readily be adduced; but the development of this theory does not come within my express views on the present occasion. It was only necessary to state thus much, in order to explain some circumstances appertaining to eruptive fevers in general, that relate especially to those points in their history which I shall presently take into particular consideration.

The eruptive fevers which I shall take into particular consideration are, *scarlatina*, *rubeola*, *variola*, and *varicella*.

Amongst the first order of symptoms of *Scarlatina*, are cough, nausea, loss of appetite, anxiety, inordinate redness of the tongue and of the throat, pain about the frontal sinuses, frequently pain in the limbs, commonly shivering, and then increased heat of the surface of the body. The febrile phenomena vary in respect to their intensity, but are generally violent in proportion to the severity of the local inflammation. The functions of the brain are also deranged, and vertigo, with disturbance of the intellects, consequently ensue.

On the third day, the face, neck, and breast, appear redder than usual; and in a few hours this redness becomes universal.

It is necessary to remark, that, in the course of this disorder, the irritation of the mucous membrane of the throat and bronchiæ takes place in the first instance; then, that of the alimentary canal; and, lastly, that of the skin.

Cases sometimes occur, in which no eruption takes place on the skin. In these instances we see the predominant characters of disorder of the gastric organs still more strongly marked: the lips and teeth are dry and crusted; the tongue is covered with a grey fur, and is red at its edges; the mucous tunic of the eye, the conjunctiva, is inflamed; there is severe pain about the epigastric region, with vomiting of green matter; frequent stools of the same appearance; great difficulty of speaking; swelling of the tonsils; and great anxiety marked in the countenance. Sometimes nervous symptoms supervene: then authors have called the disease *malignant;* and they add, that nature has not sufficient force to convey the complaint outwards; but the truth is, that the eruption does not take place, because the irritation too far predominates in the alimentary canal and bronchiæ.

On other occasions, the inflammation of the throat becomes excessive, and gangrene attacks that part.

The eyes and nostrils partake more or less of the general redness; and, in proportion to the intensity of this colour in these parts, the tendency to delirium prevails.

Disturbance of the functions of the brain is a general attendant on all severe febrile disorders, and, as it is here shown, is an effect of the local disease already described; the delirium being in a direct ratio with the inflammation of the mucous membranes; as indicated by the inflamed conjunctiva, and other evidences of this state.

If, then, we analyse scarlatina, it will appear, that the first symptoms indicate disorder of the mucous membranes of the throat, bronchiæ, and digestive organs. Hence it becomes extremely difficult, at its commencement, to determine the precise nature of the disease, as similar phenomena appear in several other forms of fever.

Whatever, therefore, may be the proximate cause of the disease, it makes its first sensible impression on the mucous surfaces, and acts secondarily upon the skin, which, of all others, would seem to be of the least importance; for persons who have the disease without any eruption, are not more liable to a second attack than those in whom the eruption has appeared; whilst the removal of the disease at its onset by emetics, exposes them to a recurrence of it. It may be remarked, that, if an emetic be administered on the commencement of the nausea, so as to remove the gastric disorder, the appearance of the cutaneous affection is often prevented. This has been commonly witnessed when scarlet fever has been epidemic, especially a few years since, when the practice of giving emetics on this occasion was very general; and experience has shown, that a person treated in this way is liable to have another attack of the disease: a fact which indicates that a certain degree of affection of the mucous membranes is necessary, in order to render them insusceptible to a recurrence of the malady.

Idiosyncrasy, and perhaps, under some circumstances, something peculiar in the degree of virulence of the contagion, may occasion a predominance of the disorder in certain parts. Hence, in some persons, the larynx and pharynx are so severely affected as to threaten suffocation; whilst, in others, the bronchiæ will be found to be principally disordered.

At another time, the inflammation attacks the cellular texture: the skin is then rendered so highly sensible, that the slightest touch occasions severe pain. In such cases, the efflorescence does not yield to pressure; and blood has been found extravasated in the cellular texture, under the cutis vera, and among the muscles.

It occasionally terminates in chronic diarrhœa, from the inflammation extending to the mucous membrane of the alimentary canal, and assuming a chronic form. Sometimes the stomach is affected in this manner; and not unfrequently the bronchiæ, when it, in some instances, gives rise to phthisis, in a manner I shall hereafter explain.

When the disorder quits the skin and mucous membranes, it not unfrequently happens that the serous membranes then take on a diseased action; and hence we find dropsies occur, as a sequel of scarlatina.

When death takes place in the height of the disease, we find the morbid appearances almost wholly confined to the mucous membranes, presenting various states, from slight inflammatory redness and tumefaction to gangrenous disorganization. The cellular membrane of the throat ordinarily participates of the inflammation of the mucous membrane of this part; and the irritation of the brain has produced sanguineous congestion, and sometimes serous effusion, in that organ.

*Rubeola.*—Measles present the same succession of phenomena as scarlatina, but they here appear in a milder form.

The early symptoms mark a disorder of the mucous membranes; such as redness of the eyes, increased secretion from the lacrymal gland, frequent sneezing, cough, and permanent pain of the frontal sinuses. The irritation of the brain which ensues, is sometimes productive of convulsions in young children.

The febrile state is sometimes violent from the beginning; but more frequently it is, for the first two days, obscure. Still it always becomes more severe before the eruption, and marks the intensity of the mucous affection.

These symptoms precede the eruption three or four days.

Between scarlatina and rubeola, there is some difference with respect to the part especially affected. In scarlatina, it is the throat; in rubeola, it is the mucous membrane of the bronchiæ. It is therefore more catarrhal, according to the language of authors. Experience has also taught us, that emetics, which are frequently capable of arresting the progress of scarlatina, do not produce the same effect in measles, nor prevent the progress of the malady, and its development on the skin, from their not acting expressly on the primary seat of the disease.

Dr. Willan mentions, that the *rubeola sine catarrho* is an unusually mild form of the disorder, which does not destroy the susceptibility to an attack in future. Two instances of its recurrence happened among his own children, at an interval of two years: and he gives us other instances, wherein the efflorescence, without fever or catarrhal symptoms, having declined, there appeared, on the fourth day from its commencement, a new efflorescence and violent disorder of the constitution.

Hence we learn, that not only the mucous membranes are the primary seat of the disorder, but that it is requisite that the affection should make an evident impression upon them, to secure the patient from a recurrence of the disease.

Sydenham recommended bleeding in measles, as the only means of checking the diarrhœa. The views we are now taking will show the propriety of this treatment; for, although the disorder, in the first stages, is chiefly confined to the mucous membrane of the bronchiæ, still, in the advanced periods of the disease, the affection occupies the mucous membrane of the stomach and small intestines; and these I have seen, after death, of a perfect scarlet colour: consequently, by reducing this inflammation, the diarrhœa will be relieved.

The succession of the phenomena from irritation of the mucous membrane, is nearly the same in rubeola and scarlatina. In both, the eruption takes place on the third or fourth day; and, when this occurs, the internal irritation nearly ceases, and consequently the violence of the fever is also alleviated.

In rubeola, the skin is not so much swollen as in scarlatina. The redness is not uniform, but arises from small spots in clusters, which do not form visible pimples, though, by the touch, they are found to be sensibly prominent. In the intervals of these spots, the skin is healthy; whilst the redness of scarlatina is everywhere the same. The skin does not attain the same degree of sensibility, nor does the cutis vera become inflamed. Neither do we find blood extravasated under it; nor that formidable degeneration into erysipelas of the face, of which scarlatina sometimes furnishes us with examples.

The cough ordinarily persists during the whole course of the fever; and this cough, with redness of the tongue, sometimes continue after the termination of the fever. During convalescence, phthisis not unfrequently makes its appearance, in those who were predisposed to that malady.

In scariatina, the corpus reticulare is more generally affected; in measles, it is the folliculous glands of the mucous membranes.

On forming a prognosis, our attention must be chiefly directed to the state of the mucous membranes. It should be favourable, as long as the disease pursues a regular course, particularly when the fever ceases on the coming-on of the eruption. When this is not the case, the predominant inflammation of the bronchiæ and alimentary canal, may create apprehensions of serious, and perhaps of fatal, consequences.

*Variola.*—This disorder attacks all the textures of the skin. The eruption presents several different characters, and is usually distinguished into *Variola discreta*, which is the mildest, and *variola confluens*, or the more inflammatory form. To these a third, which may be termed *variola maligna*, may be added, in which the inflammation of the mucous membranes is so intense as to engage so entirely the vascular energy, that no metastasis takes place to the skin, and consequently no well-marked eruption occurs. Similar circumstances sometimes happen in measles; and hence, as Hoffmann had remarked, blood-letting is often the surest means of producing the eruption in this disease.

I shall commence with *variola discreta*, because it gives us the clearest view of the malady. This form is called *discreta*, because the pustules are distinct from each other. It is also called *benign*, from the little danger attendant upon it.

The first symptoms are similar to those we find arising from an inflamed state of the mucous membrane of the alimentary canal; vomiting, with pain on pressure of the epigastrium, and the rest, common to the forms of fever already described; and, until the eruption appears, which happens on the third or fourth day, we are unable to determine positively the nature of the disorder.

The moment the eruption appears, the fever ceases, by the removal of the irritation from the interior to the skin. We no longer observe redness of the mucous membrane of the tongue, lassitude, or want of appetite; nor the convulsions, which arise from the alimentary canal being powerfully stimulated, and re-acting on the brain. But, when the eruption is at its acme, which usually occurs on the eighth day, its severity is in general sufficient to re-act on the alimentary canal, and give rise to what has been termed the secondary fever.

*Variola confluens.*—In this form, whether from peculiarity of constitution, or from the absorption of a greater quantity of miasma, is not easy to determine, though the former is the most probable cause, the pustules are more numerous, and very violent inflammatory symptoms always precede the eruption.

Generally, the intensity of the irritation of the mucous surfaces of the alimentary canal at the commencement, gives the measure of the degree of violence of the approaching cutaneous inflammation, and of the abundance of the pustules. Few have been the exceptions to this general rule.

I have also remarked, that the pustules are constantly more confluent in the more delicate region of the skin, and where the blood-vessels of the rete mucosum are most abundantly supplied with red blood, and the irritability of which is either naturally or accidentally greater. Hence the face, neck, and chest, constantly show a greater number of pustules. These are also precisely the parts which show an efflorescence, from sympathy, on the stomach being loaded with food.

I am disposed to conclude, with Sydenham, Cullen, and many others, that, in general, the confluence is in a direct ratio with the inflammatory predisposition.

In cases of extreme confluence, the primary symptoms are very severe. There is violent fever, burning heat, severe pain in the loins; sometimes vomiting, as severe as in the yellow-fever; redness of the tongue and eyes. All these symptoms indicate a severe affection of the alimentary canal. In individuals of a sanguineous habit, irritation of the lungs is sometimes superadded. By these symptoms, we perceive that the two great visceral cavities may be affected at the same time.

Towards the third or fourth day, if we observe an universal redness of the face, or eruptions very nearly approaching each other, we may prognosticate a severe and confluent disorder. From this moment we should entertain alarm, which will be increased by the persistence of the febrile symptoms.

This redness becomes covered with pustules; the progress of the development of which is nearly similar to those of variola discreta.

Towards the seventh or eighth day, the pustules become united, if they were not so from the commencement; and, when they are thus united, which principally occurs in the face, we have then no longer to deal with small insulated inflammations of the face, but with a severe erysipelas.

The face becomes enormously swollen, the eyes are closed, the mouth cannot be opened; the lips, the tongue, and the tonsils, are very much tumefied.

There is in many instances a copious salivation; and the interior of the mouth is covered with pustules, which have been traced into the ramifications of the bronchiæ.

I consider it owing to the state of the epidermis which vests the membrane in the mouth and along the bronchiæ, that the eruptions assume the pustular form in this portion of the mucous membrane. But, though these pustules are not found in other portions of the mucous membrane of the alimentary canal, those parts are the seat of an equally severe inflammation.

The facility with which red blood can penetrate the rete mucosum, also modifies in a remarkable manner the quantity of pustules: thus, they are most abundant on the face, neck, and thorax, where the rete mucosum is readily supplied with red blood. They are less abundant in the palm of the hands, soles of the feet, and along the back, where the rete mucosum is less filled with red blood; but, if rubefacients or blisters determine the blood to such parts, without at the same time breaking off the connexion with the epidermis, then they appear in abundance in these parts.

The state of the epidermis, above alluded to, on which the presence of pustules depends, is a certain degree of firmness of that membrane and of adhesion of it to the rete mucosum. Where its tenuity is extreme, and its adhesion to the rete mucosum but slight, as in the stomach and intestines, it will not admit of being elevated into distinct vesicles: it is either ruptured by the fluid effused beneath it, or detached from the subjacent membrane in an irregular manner. In the bronchiæ it is sufficiently firm and adherent to suffer it to be raised in distinct points; and Dr. Nevinson, in three cases of death from small-pox in St. George's Hospital, found pustules extending down the larynx as far as the eye could trace the minute branches of the bronchiæ. One of these specimens is in Dr. Baillie's museum.

In the slightest degrees of confluent small-pox, the fever ceases with the coming-on of the eruption, to re-appear when the pustules unite with each other. Whilst, in the more severe cases, in which the pustules are constantly united together in the face, the fever never ceases.

From what I have said, I think we may establish four degrees of variola.

1. Variola discreta; in which the fever terminates on the appearance of the eruption, and never re-appears.

2. Variola in a second degree; in which the fever terminates with the coming-on of the eruption, but returns when the pustules are at the highest degree of inflammation, which usually occurs on the eighth day.

3. Variola; in which the fever continues after the eruption, and exacerbates from the progress of the pustules, and the confusion of their inflammatory areolas.

4. Very violent; a variety in which the eruption remains incomplete, on account of the excessive predominance of the internal inflammation. It is to this variety that authors have given the name of malignant variola, which they have also given to a high degree of the confluent form.

The variola discreta is without the least danger; but this is not the case with the third and fourth species.

To these, other disorders become united; for example, an erysipelatous swelling of the face may be superadded, and raise the inflammation of the mucous membrane of the alimentary canal to its highest pitch by its re-action on it. It may even induce other disorders; and we see frequently, as a consequence, vomiting, diarrhœa, cerebral congestion, and pneumonic inflammation.

The irritation may pass the boundaries of the mucous system, and attack the cellular and serous systems: hence peritonitis, and sometimes inflammation of all the synovial membranes of the joints.

This inflammation, which is quickly established, rapidly reduces these textures to a state of suppuration.

Sometimes the patient is enabled to resist this erysipelas, and purulent abscesses form under the cutis vera, which exhale a most fetid odour.

At other times the external affection alone disappears, and death takes place, in consequence of the progression of the inflammation of the alimentary canal, characterized by what authors have denominated typhoid symptoms, by pneumonic or cerebral affection. Death takes place towards the fourteenth or sixteenth day. Antiphlogistics may retard, as stimulants will most certainly accelerate, the event.

From these premises, I think we are justified in drawing the following conclusions:

1. That the mucous membrane of the alimentary canal is the seat of the first impression of the virus.

2. That the eruption is only the secondary effect.

3. That the fever termed secondary is the sympathetic effect of the erysipelas, occasioned by the confluence of the pustules, and not the necessary consequence of the virus.

4. That all the complications depend upon a natural or acquired inflammatory or irritable disposition.

*Varicella*, is a superficial inflammation of the skin. It, like the small pox, is preceded by a febrile irritation, the characters of which are uniformly similar to those which arise from irritation of the alimentary canal.

After a slight access of fever, the papulæ make their appearance, and are attached to the skin by a pedicle.

From the first day the papulæ lose their transparency; and in about two or three days they fall off in crusts. These papulæ do not all rise at the same period: they, consequently, appear in different stages at the same time on the body.

The varicella is no preservative against the small-pox.

Although this disease is in general slight, it may still produce severe effects in persons of an inflammatory habit of body; particularly when powerful stimulants have been employed, instead of the remedies required in acute irritation of the mucous membranes of the alimentary canal.

Since, in all the eruptive diseases of which we have spoken, the affection first takes place in the interior, and afterwards on the skin, are we not justified in concluding, that the virus first attacks the viscera, and is thence transmitted to the skin?

In concluding this subject, if we may be allowed to make use of the terms direct and reverse sympathy, I think we should be enabled to make this point clear, and render more intelligible a practical illustration I shall deduce from the phenomena. For example, we should call that reverse sympathy, where the appearance of the eruption removes the disorder of the mucous membranes; and that direct sympathy, where the violence of the irritation of one of the surfaces induces a considerable degree of disturbance in the functions of the other, and is not itself alleviated by the occurrence of the secondary affection, but, on the contrary, is rendered more intense by the direct reaction of the latter.

The first we have illustrated in the variola discreta, mild state of rubeola, and erysipelas. In these examples, the sympathies being indirect, the disorder of the mucous membranes, or, in other words, fever, disappears on the coming-on of the eruption. But, in the secondary fever of small-pox, in confluent small-pox, in what have been termed gastric and bilious fevers, and generally in scarlatina, the irritation of the mucous membranes exists at the same time with that of the skin, from their direct reciprocal action on each other.

Are we not justified in asserting, that inattention to these different states has been the cause of pernicious practice, and has occasioned in fevers, as in many other disorders, a remedy to be neglected, which, if duly appropriated, might be of the greatest advantage. Thus, cold affusion applied to the skin, in cases of reverse sympathy, that is, when the internal disorder has been removed by the eruption on the skin, may occasion the re-appearance of irritation on the mucous surfaces. But, when the two surfaces act by direct sympathy, then cold affusion and ventilation will be efficacious in moderating the violence of febrile excitement; as we especially find in those fevers called gastric and bilious, and in severe forms of scarlatina.

*Sackville-street; June* 1820.

